# Personal believes - Barriers to vaccination against Covid_19

**DOI:** 10.1192/j.eurpsy.2022.1379

**Published:** 2022-09-01

**Authors:** A.H.I. Abu Shehab, A.V. Gurita, L. Luca, L.Ș. Burlea, A. Ciubara

**Affiliations:** 1 ’’Elisabeta Doamna’’ Psychiatry Hospital of Galati, Psychiatry Department, Galati, Romania; 2 ’Elisabeta Doamna’’ Psychiatry Hospital of Galati, Psychiatry Department, Galati, Romania; 3 University of Medicine and Pharmacy “Grigore T. Popa”, Psychology, Iasi, Romania; 4 University of Medicine and Pharmacy “Grigore T. Popa”, Public Health And Management In Dentistry, Iasi, Romania; 5 ’Dunerea de jos’’ University of Galati, Psychiatry Department, Galati, Romania

**Keywords:** pandemic, Immunization, mental health, Vaccine refusal

## Abstract

**Introduction:**

The ongoing global pandemic of Covid_19 had a huge pressure to accelerate the development process of Covid_19 vaccine. This acceleration of the vaccine appearance raised many concerns regarding the effectiveness and the adequate safety of the vaccine among general population.

**Objectives:**

The aim of the study is to determine the reasons behind vaccine refusal among general population.

**Methods:**

Online questionnaire with the subjects’ agreement; The study included 61 participants aged between 18 and 40 years old. The study was effectuated in October 2021.

**Results:**

Most of the participants (n=60, 98.36%) declared that they knew some persons who have refused the Covid_19 vaccine. Moreover, a number of 29 participants (48.33%) declared that vaccine refusal among the people who refused the vaccine was due to personal believes.

**Conclusions:**

The success of the vaccination programs mainly depends on the proportion of the population that receive the vaccine. It is crucial to implement new strategies to increase the acceptability of Covid_19 vaccine.

**Disclosure:**

No significant relationships.

